# Analysis of the Mechanical Properties of Polymer Composites Reinforced with Charcoal Particulate

**DOI:** 10.3390/ma18122746

**Published:** 2025-06-11

**Authors:** Josinaldo O. Dias, Rayara Davel Siqueira, Bruno Fonseca Coelho, Amanda O. Conceição

**Affiliations:** Agricultural Sciences and Engineering Center, Federal University of Espírito Santo, Alegre 29500-000, Brazil; rayara.siqueira@edu.ufes.b (R.D.S.); bruno.coelho@edu.ufes.br (B.F.C.); amandaoliv41@gmail.com (A.O.C.)

**Keywords:** HDPE, charcoal, bio-reinforcement circular economy

## Abstract

Naturally reinforced polymer composites have emerged as a promising sustainable alternative to conventional polymers due to their biodegradability. This study aimed to develop a composite by incorporating charcoal particulate into a recycled high-density polyethylene (HDPE) matrix and evaluating its mechanical properties. Two manufacturing methods (compression molding and extrusion) and four charcoal concentrations (0, 5, 10, and 15%) were investigated. Characterization involved tensile tests and non-destructive evaluation using wave propagation and ultrasound techniques. The experiment followed a completely randomized design with a 4 × 2 factorial arrangement, comprising eight treatments. Statistical analysis was conducted using Tukey’s test for multiple comparisons. The tensile test results indicated that the manufacturing methods of compression molding and extrusion led to significant differences in the elastic modulus (MOE) variable, in contrast to the results observed for the maximum stress variable. However, the addition of charcoal particulate caused a notable reduction in maximum tensile strength (approximately 50%), from 20.17 to 11.19 MPa, and a 22% decrease in the MOE, from 310.93 to 242.88 MPa, compared to unreinforced HDPE. Non-destructive testing confirmed the tensile test findings, also indicating a reduction in MOE. Despite the decline in mechanical properties, these composites remain viable for applications prioritizing lightweight structures, thermal insulation, or chemical resistance. Furthermore, their use enhances the valorization of waste and increases sustainability by reducing environmental impact.

## 1. Introduction

Polymeric composites, defined as multifunctional and highly versatile materials, have progressively gained prominence in the field of construction materials and are widely applied in industries such as automotive, aerospace, biomedical, and civil engineering. The versatility of these composites, demonstrated by their ease of processing, flexibility, and favorable mechanical properties [1], establishes the development of polymer matrix composites as one of the most effective strategies for modifying and enhancing the characteristics of polymers.

The insufficient mechanical properties of pure polymers, particularly in terms of strength and stiffness, limit their applicability in various structural applications. The incorporation of reinforcements, such as particles, continuous or discontinuous fibers, and flakes, enables the modulation of the properties of polymeric composites, making them more suitable for a wide range of applications [2,3].

The incorporation of reinforcements into polymer matrices is a widely used strategy to enhance the properties and performance of polymeric composites. These reinforcements, when integrated into the matrix, increase the material’s volume, reduce production costs, and improve mechanical and physical properties, such as strength, stiffness, and dimensional stability [4].

Given the success of using fibers for the production of composites, science has proposed the utilization and study of new bio-reinforcements with sustainable characteristics. Among these is charcoal, a material composed almost entirely of carbon, highly porous, and with irreversible adsorption capacity [5,6].

Charcoal particulates stand out as potential particles for reinforcing polymer matrix composites. Recent studies [7,8,9,10,11] have demonstrated the potential of charcoal as a renewable, low-cost resource with physicochemical properties suitable for various applications. This suitability arises from its high carbon concentration, high porosity, large surface area, and irreversible absorption capacity. Moreover, charcoal fines exhibit properties similar to those of graphene, graphite, and diamond, but with the advantage of a simpler and economically viable production process [12,13].

The incorporation of particles into polymeric matrices has been widely used to enhance the mechanical properties of composites, such as tensile strength and elastic modulus [14]. Husseinsyah and Mostapha [15] investigated the effects of adding coconut shell particles on the mechanical, water absorption, and morphological properties of polyester-based composites. The composites were prepared with different particle contents, ranging from 0% to 60%, in a polyester resin matrix. The results showed that increasing the particle concentration improved the tensile strength, Young’s modulus, and water absorption capacity, while the elongation at break was reduced.

Eze et al. [16] investigated the mechanical properties and flame-retardant behavior of low-density polyethylene (LDPE) composites reinforced with bamboo powder particles. The results indicated that an increase in particle content led to a reduction in tensile strength and elongation at break, while the stiffness of the composites was significantly enhanced.

In light of this potential, it is essential to analyze the mechanical properties of composites reinforced with charcoal fines in order to understand their structural performance and behavior under different processing conditions. Furthermore, the evaluation of the effects of functionalizing the charcoal particles, as well as the investigation of new combinations between polymer matrices and reinforcing particles, is crucial for the development of advanced materials with specific properties, suitable for various applications.

This study presents a novel approach by developing and characterizing a sustainable composite combining two waste-derived materials: recycled HDPE and charcoal fines. This approach aligns with circular economy principles and adds unique value from both environmental and material science perspectives.

## 2. Materials and Methods

### 2.1. Charcoal Particle Production

For the production of charcoal, Eucalyptus sp. wood residues were used. The wood samples were initially ground using a Tigre A4 hammer mill (Moinhos Tigre, São Paulo, Brazil) for 2 min per 500 g of biomass. Subsequently, they were processed in a Willy TE-650 knife mill, achieving a sawdust particle size of approximately 50 mesh. This step is crucial, as the smaller biomass particle size enhances heat transfer during pyrolysis, resulting in a higher yield of charcoal fines.

The ground wood samples were dried in an oven at 102 ± 2 °C for 20 h. The dried material was then pyrolyzed in an electric muffle furnace, reaching a final temperature of 800 °C, with a heating rate of 5 °C min^−1^ and a residence time at the final temperature of 60 min.

To ensure a homogeneous distribution of carbon particles within the polymer matrix, pilot tests were conducted to identify the optimal particle size for the matrix used in this study. For this purpose, the particles underwent a classification process, selecting those that passed through a 40-mesh (0.425 mm) sieve and were retained on a 60-mesh (0.250 mm) sieve. The selection of particles within this size range aimed to minimize agglomeration and ensure a uniform dispersion of the reinforcement in the matrix, promoting better interaction between phases and, consequently, enhancing the composite material’s properties.

### 2.2. Production of the Composites

The recycled HDPE was obtained through a partnership with the Association of Recyclable Material Collectors and collection efforts carried out in communities in the city of Jerônimo Monteiro, ES. Product packaging was collected and cleaned with water and neutral detergent to remove residues, impurities, and contaminants. The cleaned packaging was manually cut into smaller pieces using standard scissors. The cut pieces were then ground in a knife mill to reduce the particle size to 18 mesh (1.00 mm).

To assess the impact of different production methods and the incorporation of charcoal particulate into the polymeric structure, two manufacturing processes were defined, and specific fine proportions based on weight were established for the experimental design (Table 1).

The composites were produced at the Department of Chemistry (DEQUI) of CEFET-MG, located in Belo Horizonte, Minas Gerais, through thermal processing using pressing and extrusion methodologies. For both methods, material preparation was conducted to ensure better homogenization of the matrix and bio-reinforcement. The samples were prepared in an M.H. Equipment homogenizer, model MH-100. The mixture was maintained in the chamber for 25 s under a maximum rotation of 100 rpm. After this step, the material was granulated in a KIE knife mill, model MAC250BX, for about 1 min. Finally, the process of producing the composite materials was initiated.

**Figure 1:**
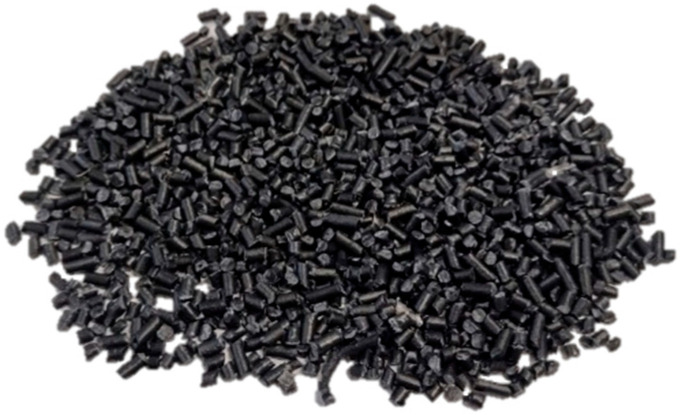
Pelletized material after the extrusion process.

For the extrusion process, a single-screw extruder from THERMO SCIENTIFIC, model HAAKE POLYLAB QC (Thermo Fisher, Waltham, MA, USA), with four heating zones, was used. The homogenized and mixed sample was fed into the extruder using the following parameters: (i) screw rotation speed of 20 rpm; (ii) temperature profile set to 160 °C for heating zones 1, 2, and 3; and (iii) 170 °C for zone 4 (exit or die zone). After processing, the filaments were pelletized (Figure 1) using an Axplástico laboratory pelletizer (AXPlástico, Diadema, Brazil).

The pelletized material was pressed into sheets approximately 150 × 100 × 1.4 mm in size using a hydraulic press with heating, model SL11 by SOLAB (SOLAB, Piracicaba, Brazil), up to 3 MPa. The pellets were placed in a mold and pressed for 10 min at 180 °C.

**Figure 2:**
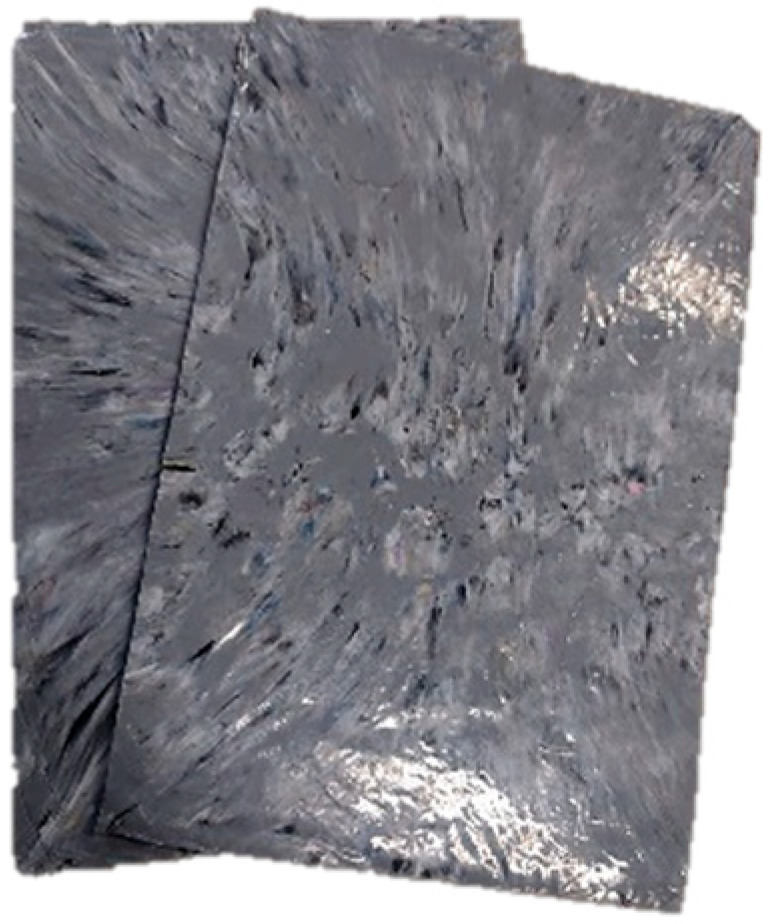
Boards produced by pressing process.

For the pressing method, procedures similar to those applied to the pellets from the extrusion process were adopted, but using only the homogeneous mixture without passing through the extruder. After the pressing stage, the plates (Figure 2) were removed from the press and subjected to a 5-min cooling period at room temperature.

### 2.3. Determination of Mechanical Properties of Composites

#### 2.3.1. Scanning Electron Microscopy (SEM)

After the flexural test, the fracture region morphology of the specimens from each condition (0, 5, 10, and 15% fine charcoal particles) was evaluated using SEM images. The specimens obtained from the tensile test were cut into 1 cm pieces to fit the sample holder of the equipment. The samples were fixed onto the appropriate sample holder using double-sided carbon conductive tape to establish electrical contact between the sample and the holder, aided by carbon-based paint, and were gold-coated for 10 s. Finally, micrographs were obtained using a JEOL JSM 840A scanning electron microscope (LabWrench, Midland, ON, Canada), operating with an electron beam at an acceleration voltage of 10 kV and a current of 6 × 10^−11^ A.

#### 2.3.2. Tensile Test

The tensile test was conducted at the Wood Anatomy Laboratory, Department of Forest and Wood Sciences, UFES, located in Jerônimo Monteiro. The test specimens were evaluated using an EMIC mechanical testing machine, model DL3000. A 10 kN load cell was used.

The dimensional characterizations (length and width) of the specimens were performed using a digital caliper MTX, model 316119 (Tools World, Itajaí, Brazil), with a resolution of 0.01 mm. For thickness measurements, a Mitutoyo micrometer, model MDC-SX (Mitutoyo, Suzano, Brazil), with a graduation of 0.001 mm and accuracy of ±0.002 mm, was used. The mass was determined using a Weblaborsp precision balance, model S2202 (Weblabor, Mogi das Cruzes, Brazil), with a reproducibility of 0.01 g and linearity of ±0.1 g.

The test followed the technical standard ASTM D638-22 [17]—Type IV.

The specimens were prepared using the cold molding process. For this, a metallic mold with dimensions of 115 × 20 mm was positioned on the surface of the plate and subjected to manual compression in a press. The compression load was applied until the mold completely cut through the plate. Subsequently, the mold was removed, allowing the specimen to be obtained.

The test was conducted at room temperature, and the results were derived from the average of nine specimens tested for each material composition across the different composite production processes.

The modulus of elasticity (E) was determined by the slope of the straight line fitted to the linear segment of the initial stress–strain curve.

#### 2.3.3. Propagation Testing for Excitation

The wave propagation testing for excitation was conducted using the SONELASTIC device (Sonelastic, Ribeirão Preto, Brazil), following the guidelines of the ASTM E—1876 [18] and ASTM C—215 [19] standards under flexural boundary conditions. This is a non-destructive technique for determining the elastic moduli and damping of materials through the natural vibration frequencies of the specimen.

**Figure 3:**
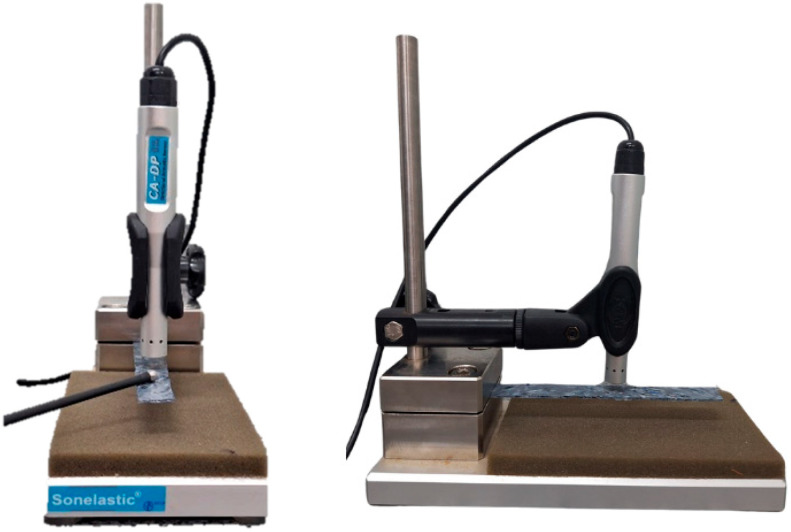
Impulse excitation technique used in the determination of dynamic modulus of elasticity (MOE d) through the propagation of excitation waves.

For the experiment, a microphone was used to capture the vibration caused by the impact application, with a fixture in the form of a ruler (Figure 3) to support the specimen. Through a manually applied mechanical impulse excitation (a light “tap” on the specimen), the software is responsible for acquiring the acoustic response, processing the signal, detecting the natural frequencies, and performing the calculations for the dynamic modulus of elasticity. For the calculations, it is necessary to provide the mass, dimensions, and corresponding uncertainties of the specimen.

For the execution of the test, 11 specimens were used for each material composition and for each of the manufacturing processes adopted, with dimensions of 140 mm in length, 15 mm in width, and 1.4 mm in thickness.

#### 2.3.4. Ultrasonic Wave Propagation Test

The ultrasonic wave propagation test was conducted using the Ultrasonic Timer FAKOPP equipment (Fakopp, Sopron, Hungary), in accordance with the guidelines established by the NBR 58000 (2007) standard [20]. For the experiment, two piezoelectric transducers, model SD 33 (Fakopp, Sopron, Hungary), were employed. These transducers were coupled to the cross-sections of the specimen using a clamp, operating at a frequency of 90 kHz.

For the experiment, 11 rectangular specimens (SP) were used, each with dimensions of 140 × 15 × 1.4 mm (length, width, and thickness) for each proportion of charcoal particulate evaluated, as well as for each composite production method. These same specimens were used in the excitation wave propagation tests.

The measurements were taken along the length of the specimen, recording the propagation time of the wave in microseconds. Based on the obtained values for the propagation time and the distance traveled by the wave, corresponding to the length of the specimen, the wave propagation velocity is calculated.

The propagation velocity (V) is calculated using the Equation (1).

(1) $$V=\frac{L}{\left(t*10^{-6}\right)}$$

where V is the wave propagation velocity (m/s), L is the length of the specimen (m), and t is the wave propagation time (µs).

The travel time (t) was subtracted by 37 µs as a correction factor to compensate for the transit time of the transducer within the launch and reception probes. From the propagation velocity, the dynamic modulus of elasticity (MOE d) was calculated using Equation (2).

(2) $$MOE\;d=V^{2}\times \rho \times 10^{-6}$$

where MOE d is the dynamic modulus of elasticity (MPa), V is the wave propagation velocity (m/s), and  ρ   is the material density (kg/m^3^).

The experiment was conducted using a completely randomized design, arranged in a 4 × 2 factorial layout (coal particulate proportions × manufacturing process), resulting in a total of 8 treatments. Initially, statistical assumptions were tested, including the Shapiro–Wilk test to assess normality and variance homogeneity, both considering a significance level of *p* < 0.05. After validating the assumptions, the data were analyzed through analysis of variance (ANOVA), using the F-test to detect significant differences (*p* < 0.05). For mean comparisons, the Tukey test was applied, also with a significance level of *p* < 0.05.

## 3. Results and Discussion

**Figure 4:**
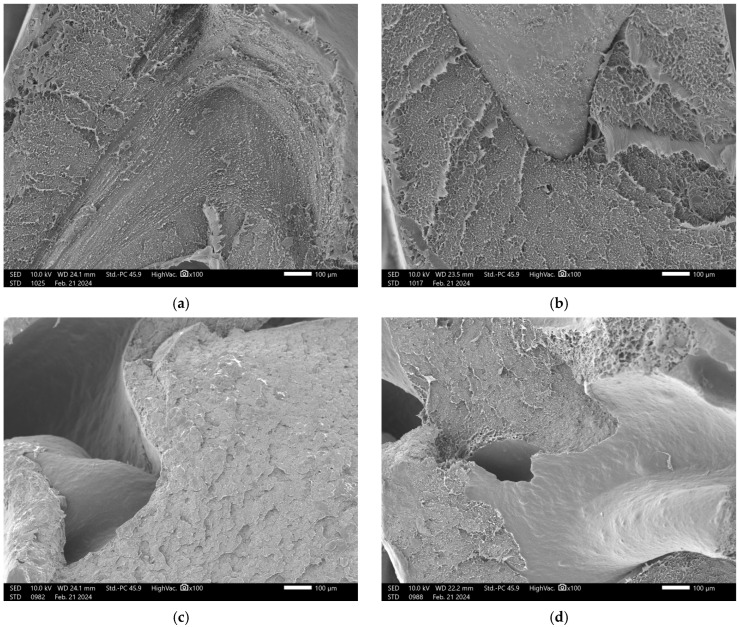
Micrograph of HDPE/charcoal fines composites using the extrusion method, at 2000× magnification: (**a**) T1-100:0; (**b**) T2-95:5; (**c**) T3-90:10; and (**d**) T4-85:15.

Figure 4 presents micrographs of the fracture region of composites containing different proportions of charcoal fines, produced by the extrusion method, obtained at 100× magnification. Micrographs A, B, C, and D correspond to treatments with 0, 5, 10, and 15% charcoal particles, respectively.

It can be observed that with the increasing addition of charcoal particles (Figure 4c,d), there is a non-uniform distribution of the particles within the high-density polyethylene (HDPE) matrix. This behavior is evidenced by the striated and rough morphology, with deep valleys and some coarse ridges, where the stretching of the plastic material becomes clearly visible.

The particle pull-out, highlighted by red circles, indicates the presence of a weak filler–matrix interface, which limits the mechanical performance of the composite. The evidence of particle detachment suggests that the adhesion at the interface was insufficient to withstand the stresses generated during fracture, which may impact the reduction of the tensile strength and the modulus of elasticity (MOE) of the composites.

This weak interface compromises the efficiency of the material reinforcement. Previous studies, such as [21], indicate that smaller particles tend to enhance the interaction between the matrix and the reinforcement. However, this effect was not observed in the present study, possibly due to the broad particle size distribution (0.425 mm > 0.250 mm) and the high particle concentration in the analyzed treatment.

The physical interpenetration of the particles with the matrix, as evidenced by the observed detachment, suggests the need to improve interfacial adhesion in these composites. The incorporation of coupling agents could be an effective strategy to strengthen the interface and enhance the performance of the composites.

A coupling agent is a chemical compound that acts at the interface to create a chemical bridge between the reinforcement and the matrix. It improves interfacial adhesion when one end of the molecule anchors to the reinforcement surface, while the functional group at the other end reacts with the polymeric phase [22].

**Figure 5:**
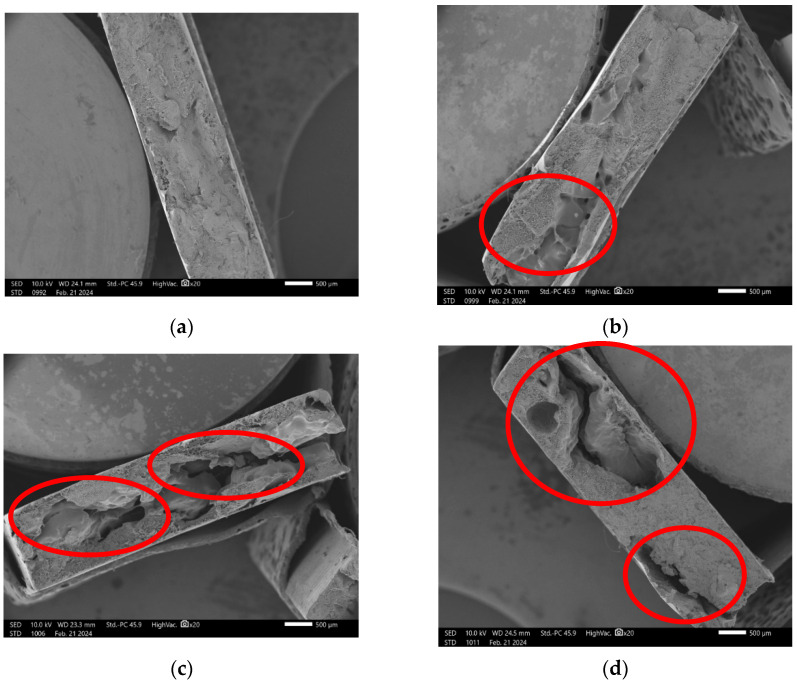
Micrograph of HDPE/charcoal fines composites using the pressing method, obtained at 20× magnification: (**a**) T1-100:0; (**b**) T2-95:5; (**c**) T3-90:10; and (**d**) T4-85:15. Red circles highlight voids formed at the fracture surface.

Figure 5 presents micrographs of the fractures of composites containing 0%, 5%, 10%, and 15% charcoal fines, obtained at 20× magnification, of the composites produced by pressing. Image a shows the micrograph of the fracture in the absence of charcoal fines. A smooth surface is observed, characterized by the absence of visible cracks or irregularities. Images b, c, and d, corresponding to treatments T2, T3, and T4, containing 5%, 10%, and 15% fines, respectively, highlight an increase in the presence of voids (marked by red circles) as the concentration of charcoal fines increases, regardless of the manufacturing method employed.

According to Marinelli et al. [23], the presence of voids in the polymeric matrix, in concentrations exceeding 20% of the total volume, significantly compromises the mechanical properties of composite materials. The reduction in maximum stress and the modulus of elasticity (MOE) observed in composites with higher fine charcoal content suggests that the formation of voids may have been the determining factor for this decrease.

From the tensile test, it was possible to obtain the results for maximum stress, modulus of elasticity (MOE), and rupture strain of the composites manufactured by pressing and extrusion methods, and with different percentages of incorporation of charcoal particulate.

The obtained results allowed for the evaluation of the applicability of the composites based on their mechanical properties.

The comparison between the production methods (pressing and extrusion), conducted through analysis of variance followed by the Tukey test (*p* < 0.05), did not identify statistically significant differences in the maximum tensile stress of the composites, as presented in Table 2.

On the other hand, the variation in the proportion of charcoal particulate significantly influenced the maximum stress, as shown in the data presented in Table 3.

The application of the Tukey test revealed that the treatment composed solely of HDPE, subjected to the pressing process, exhibited significantly different averages when compared to the other treatments that included the incorporation of particulate. However, for the composites produced by extrusion, the test did not detect statistical differences between treatments 1 and 2 (which contained 0% and 5% charcoal particulate, respectively), nor between treatments 2, 3, and 4 (which contained 5%, 10%, and 15% charcoal particulate, respectively).

The tensile test revealed that the composite made exclusively from high-density polyethylene (HDPE), without the incorporation of coal particulate, exhibited a maximum rupture stress 19% higher when manufactured by the pressing method compared to extrusion processing. When comparing these results with the reference values for HDPE, reported by Peacock [24], Mulinari et al. [25], and Saleh et al. [26], which range from 34 to 16.7 MPa, it is observed that the values obtained in this study fall within the expected range.

The incorporation of coal particulate into the composites resulted in a reduction in the maximum stress observed, regardless of the processing method used (pressing or extrusion). In the composites manufactured by pressing, the maximum stress decreased by approximately 50% when comparing treatment 1 (100% HDPE) with treatment 4 (85% HDPE and 15% coal particulate). On the other hand, in the composites obtained by extrusion, the reduction in maximum stress was around 43%.

As evidenced by Rosa [27], the tensile strength analyses demonstrated that the addition of biomass from acai peel to high-density polyethylene (HDPE) resulted in a significant reduction in mechanical properties, particularly in maximum stress, when compared to pure HDPE.

The statistical analysis of the elasticity moduli of the composites, using Tukey’s test, indicated a significant difference between the pressing and extrusion manufacturing methods (Table 4).

The values of the static modulus of elasticity (MOE) of the composites produced by the pressing method showed a marginal increase compared to those produced by the extrusion method. In treatments without the incorporation of fine particles, the composites obtained by pressing achieved a static MOE of 315.26 MPa, representing an increase of approximately 2.7% compared to the composites manufactured by extrusion, which recorded an MOE of 306.6 MPa (Table 5).

Although exhibiting a lower modulus of elasticity (MOE) compared to the composites made by pressing, the composites obtained by extrusion displayed a higher standard deviation among the analyzed samples.

In relation to the incorporation of coal particulate, it was found that the static modulus of elasticity (MOE) exhibited behavior similar to that observed for the maximum stress, as determined in the tensile test. Both variables did not show statistically significant differences between the treatments that included reinforcement with coal particulate. Furthermore, a trend of reduction in the values of these variables was observed as the percentage of added particulate increased.

Table 5 demonstrates that the increase in the concentration of charcoal particulate in the composites is correlated with a significant reduction in the modulus of elasticity. The incorporation of 15% charcoal particulate resulted in a 12.3% reduction in the modulus of elasticity for the composites produced by the pressing method and a 31.8% reduction for those obtained through extrusion, compared to pure high-density polyethylene (HDPE) without the addition of particulate. These results show that the addition of charcoal particulate compromises the material’s ability to withstand deformation before fracture, leading to a degradation of the mechanical properties.

**Figure 6:**
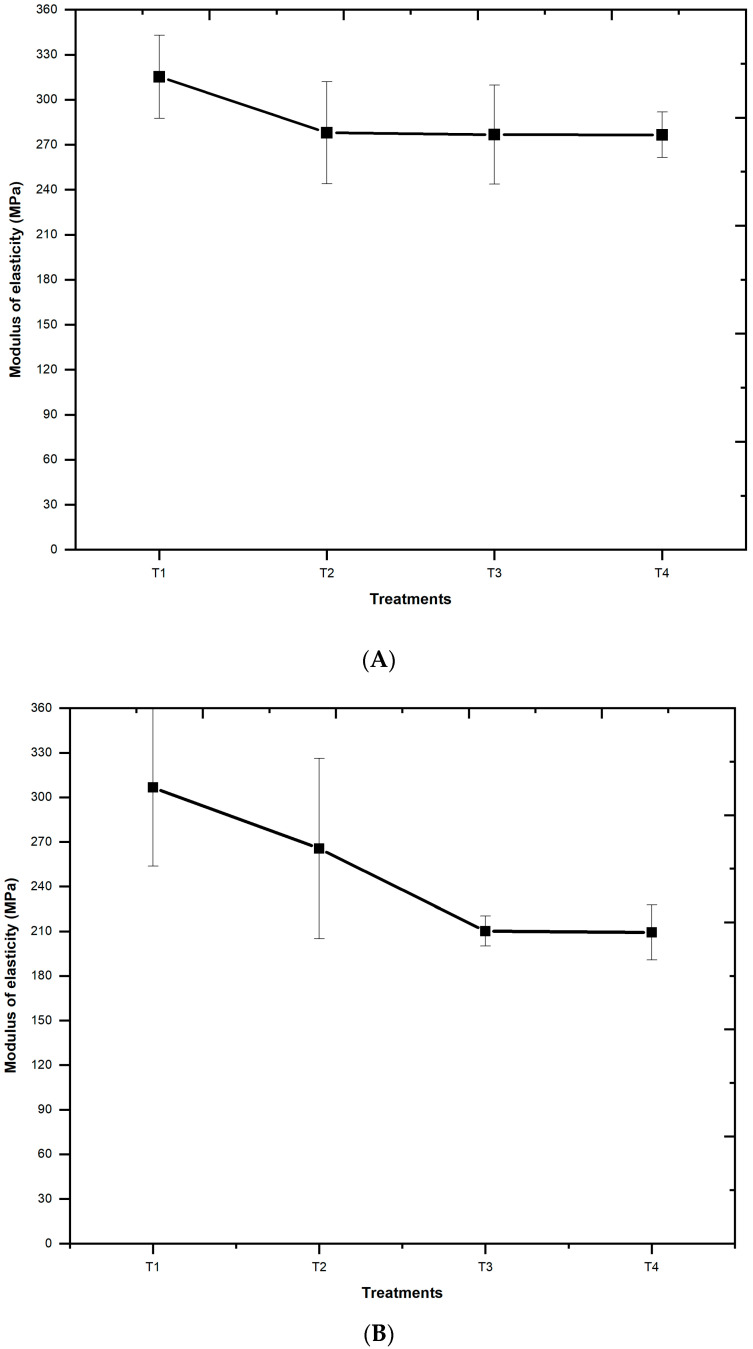
Trend graph of the variation of static modulus of elasticity (MOE) for composites produced by the methods of: (**A**) pressing and (**B**) extrusion.

The modulus of elasticity values was analyzed through graphs to assess the trend of variation as a function of the different additions of charcoal particulate to the HDPE matrix. The outcome of this analysis is presented in Figure 6.

The results obtained in this study corroborate the findings of Melo [25], which demonstrated that the addition of shell powder particles to high-density polyethylene (HDPE) composites leads to a significant reduction in the modulus of elasticity. Melo [28] determined the modulus of elasticity of pure HDPE to be 560.91 ± 1.70 MPa.

Ayadi et al. [29] investigated HDPE composites reinforced with rice husk biochar and wood flour. The authors observed that the biochar obtained through rapid pyrolysis at temperatures above 400 °C reduced the mechanical properties of the composites. This difference was attributed to the distinct chemical compositions and surface structures of the reinforcements analyzed.

Studies by Das et al. [30] and Khan et al. [31] highlighted a non-linear relationship between the tensile strength of composites and the concentration of biochar. Beyond a specific threshold, increases in biochar concentration significantly reduced tensile strength, accompanied by a transition in the matrix behavior from ductile to brittle. This behavior was attributed to particle stacking and polymer matrix crosslinking at high biochar concentrations, which promote brittleness and reduce the material’s load-bearing capacity.

Additionally, a concomitant reduction in tensile strength and percentage elongation was observed with increasing biochar concentration. These results indicate that the composite’s stiffness increases as its ductility decreases, underscoring the need to determine an optimal biochar concentration to balance these properties [8]. Such behavior is often associated with factors like the formation of interfacial voids and the disruption of polymer matrix continuity.

Awad et al. [32] emphasized that the mechanical performance of HDPE composites reinforced with particles is influenced by parameters such as particle size, the matrix-reinforcement interface, and particle dispersion. The interface plays a crucial role in load transfer between the matrix and reinforcement, directly affecting the composites’ strength and toughness [33]. However, at high particle concentrations, agglomeration occurs, hindering uniform distribution and compromising the efficiency of the interface.

The strength of composites is determined by the critical fracture path. While hard particles can concentrate local stresses and promote crack initiation [34], they can also act as barriers to fracture propagation, thereby increasing toughness [35].

In the context of polyester composites reinforced with charcoal particles, Akaluzia et al. [12] reported that an increase in reinforcement content led to insufficient interfacial adhesion, favoring the formation of microcracks and reducing resistance to crack propagation. This behavior was attributed to stress concentration and the formation of structural voids, which decreased the energy required for fracture.

Dalpiaz [36] demonstrated that particle size directly affects the tensile strength of composites. As particle diameter decreases, the surface area increases, enhancing matrix-reinforcement interaction and reducing stress concentrations. This effect results in higher tensile strength.

The observed reduction in mechanical properties in this study can be explained by the low efficiency of stress transfer between the matrix and the particles, likely due to inadequate interfacial interaction. Fu et al. [34] emphasized that in systems with weak interfacial adhesion, discontinuities caused by delamination significantly reduce load transfer. In contrast, well-bonded interfaces promote synergy between matrix and reinforcement, increasing composite strength.

Zhao et al. [37] highlighted that reducing particle size increases the particle–matrix interfacial area, promoting better load transfer and higher strength. This effect is more pronounced for larger particles, suggesting the existence of a critical size below which further reductions in size have a limited impact on the mechanical properties of the composite.

Another explanation for this type of behavior can be attributed to the physical characterization of the composite. The addition of particulate to the matrix may have led to an increase in the porosity of the material, resulting in composites with more defects and imperfections. As a consequence, the material’s strength is reduced.

In their study, Das et al. [38] observed that the addition of more than 4% by weight of coal filler to polyester composites resulted in a reduction of mechanical properties, characterized by a decrease in strength. The authors attributed this decrease to the aggregation of coal particles in the polymer matrix, which hinders stress transfer and the formation of a complex interpenetrating network, which can act as a crack initiator.

Although the composites exhibited a reduction in mechanical properties, it is important to consider that other characteristics, beyond mechanical strength, may be relevant for specific applications. The performance of composites is multifaceted and depends on the particular application. The reduction in mechanical properties does not rule out the use of these materials in components where the design can compensate for this limitation by exploiting other features such as lightness, thermal insulation, or chemical resistance.

The results obtained from the excitation wave propagation test demonstrate the influence of the coal particulate content and the production method on the dynamic modulus of elasticity (MOE d). The statistical analysis, conducted using the Tukey test, revealed no significant differences in the dynamic modulus of elasticity (MOE d) between the pressing and extrusion production methods (Table 6), which contrasts with the findings for the static modulus of elasticity (MOE e) assessed in the tensile test.

The statistical analysis, using the Tukey test, demonstrated that the coal particulate content exerts a significant influence on the dynamic modulus of elasticity (MOE d) of the composites (Table 7). Treatments 3 and 4, corresponding to 10% and 15% particulate, respectively, showed no statistically significant differences, aligning with the results observed for the static modulus of elasticity (MOE e).

The results demonstrated that the composite without the incorporation of charcoal particulate exhibited the highest dynamic elastic modulus (MOE d). The progressive addition of charcoal particulate to the polymer matrix promoted a systematic reduction in MOE, a behavior also observed in the tensile test.

The decrease in mechanical strength observed with the increased addition of charcoal reinforcement can be attributed to the reduction in the interfacial area between the matrix and the filler. The increase in the concentration of reinforcement particles restricts the polymer matrix’s ability to diffuse and efficiently adhere to each particle, compromising load transfer and, consequently, the mechanical properties of the composite [32].

The dispersion of particles in the matrix significantly influences the composite’s elastic modulus, as highlighted by Lins [39]. According to the author, uniform dispersion increases the interfacial contact area, favoring load transfer and, consequently, the modulus. The decrease in the observed dynamic elastic modulus can be explained by the presence of particle clusters or weak adhesion between the matrix and the reinforcement, both of which compromise load transfer and reduce the modulus.

The low interfacial adhesion, possibly attributed to incompatibility between the components, limited chemical interaction, or the formation of pores and voids resulting from the presence of the reinforcement, may have been a critical factor in the observed property reduction. Previous studies [40,41] suggest that the application of coupling agents or surface modification of the particles are effective strategies to improve interfacial adhesion and, consequently, the properties of composites.

A similar finding was reported by Pauleski et al. [42], who investigated the feasibility of using rice husk and wood particles in the manufacture of composites, utilizing high-density polyethylene (HDPE) as a binder. The results demonstrated that, with the decrease in the percentage of rice husk in the mixture, the values of the elastic modulus (MOE) increased, regardless of the proportion of HDPE used.

During their wave propagation, waves interact with the heterogeneities of the structure, such as interfaces between distinct materials, defects (voids, cracks, delaminations), geometric variations (holes, reinforcements), and boundaries with the external environment. These interactions result in phenomena such as reflection, refraction, diffraction, and attenuation of the incident wave, depending on the material properties and the geometry of the discontinuity [43].

One of the objectives of the ultrasonic wave propagation test, performed using an ultrasonic device, is to generate different types of waves in a specific material, analyzing its structural characteristics and detecting defects based on how each type of wave propagates [44,45].

The average values of density, ultrasonic wave propagation velocity, and dynamic modulus of elasticity (MOE d) determined through the ultrasound test are presented in Table 8. Data analysis revealed that the production methods of pressing and extrusion exhibited statistically significant differences in the variables of ultrasonic wave velocity and dynamic modulus of elasticity (MOE d), while no significant difference was observed for the density variable.

To analyze the incorporation of particulate, Table 9 shows that the addition of these materials caused significant changes in density properties and dynamic modulus of elasticity (MOE d). A trend of reduction in dynamic MOE, density, and ultrasonic wave velocity of the composites was observed as the proportion of added coal particulate increased.

The average density values observed for the treatment without the addition of particulate were 875.5 kg/m^3^ for the composites obtained by pressing and 929.3 kg/m^3^ for those produced by extrusion. The incorporation of particulate resulted in a reduction of approximately 5% in the density of the composites produced by pressing and around 19% in those produced by extrusion.

The dynamic modulus of elasticity (MOE d) did not show significant differences between the composites manufactured by pressing with the addition of particulate. For the composites produced by extrusion, it was observed that treatments 3 and 4, containing 10% and 15% coal particulate, respectively, showed no statistically significant differences between them. These results were consistent for both the elasticity moduli obtained by tensile testing and those determined by wave propagation excitation tests.

In accordance with the findings of this study, Karmarkar et al. [46], in a comparative analysis of the dynamic and static elasticity moduli of HDPE composites reinforced with natural fibers, reported that as the fiber content increased, the ratio between the dynamic modulus and the tensile modulus significantly decreased. This reduction was attributed to the stiffening of the polymer by the incorporated fibers.

It was also observed that the values of the dynamic elasticity modulus obtained through ultrasound were higher than those of the static modulus determined by tensile tests. This difference, also reported in other studies [47,48], can be attributed to the viscoelastic behavior of the material. The composites exhibit both elastic and viscous deformations simultaneously, leading to an overestimation of the dynamic modulus relative to the static modulus, as highlighted by Borůvka et al. [49].

Table 10 and Table 11 present the Pearson correlation matrix, indicating the correlation coefficients and their respective significance values (*p*-values) for all the variables analyzed based on data obtained from ultrasonic wave propagation tests conducted via pressing and extrusion methods, respectively. The results for the composites produced by the pressing method revealed correlation coefficients of 0.310, 0.660, and 0.826 for the combinations of Ultrasonic Wave Velocity (UWV) × Density (ρ), Modulus of Elasticity (MOE) × Density (ρ), and Modulus of Elasticity (MOE) × Ultrasonic Wave Velocity (UWV), respectively.

The composites produced by the extrusion method revealed correlation coefficients of 0.397, 0.850, and 0.839 for the combinations of Ultrasonic Wave Speed (UWS) × Density (ρ), Modulus of Elasticity (MOE) × Density (ρ), and Modulus of Elasticity (MOE) × Ultrasonic Wave Speed (UWS), respectively.

These results indicate the presence of strong positive and statistically significant correlations between the variables, suggesting the existence of a direct linear association between them. This means that as one variable increases, the others tend to increase as well. The upper diagonal represents the significance values. The lower diagonal represents the obtained values.

The results of this study are in accordance with those found by Nogueira and Willan [50], who established a direct relationship between the propagation speed of ultrasonic waves and mechanical properties such as elastic modulus, Poisson’s ratio, and material density. These authors emphasized that, although these properties are not directly linked to the material’s strength, they significantly influence wave propagation within the composite medium.

The introduction of coal particles into the matrix may result in an increase in the material’s porosity. This increase in porosity, in turn, may lead to a decrease in the composite’s density, negatively impacting the propagation of ultrasonic waves. The presence of pores acts as structural defects, scattering and attenuating the ultrasonic waves, which results in a reduction in propagation speed. The lower density of the porous material reduces the resistance to wave propagation, facilitating their transmission.

These results are consistent with studies by Toyama et al. [51] and Mardanshahi et al. [52], who also identified that matrix cracking and the consequent damage caused decreased the stiffness of the composites, leading to a reduction in wave propagation speed.

**Figure 7:**
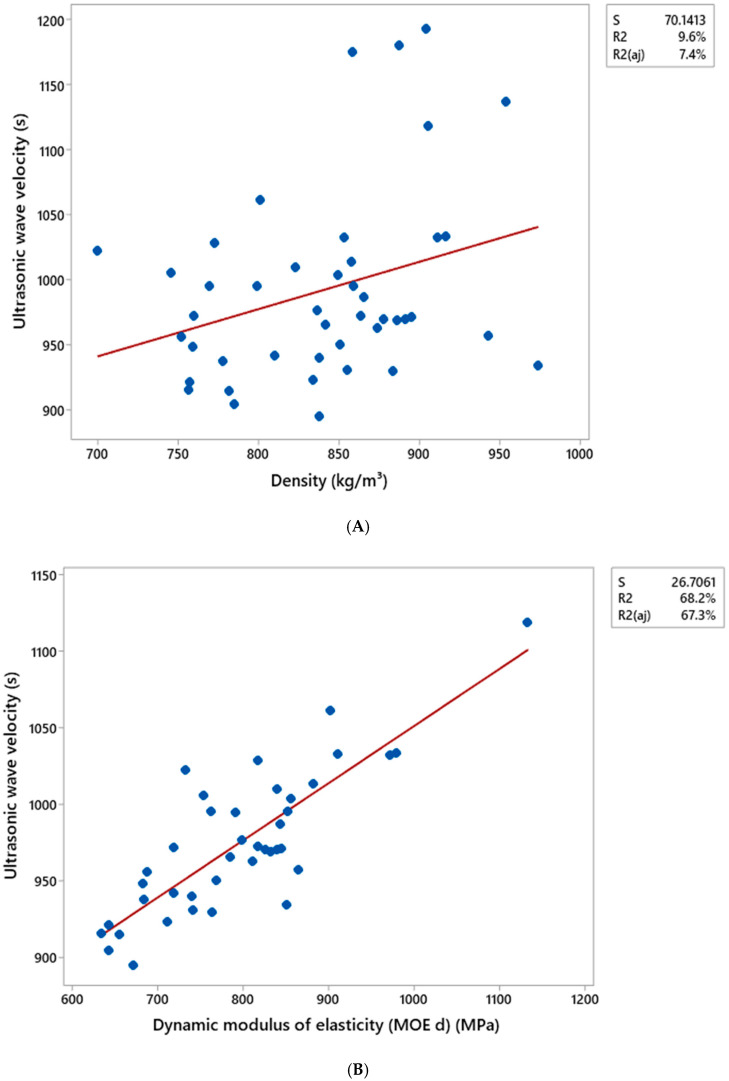

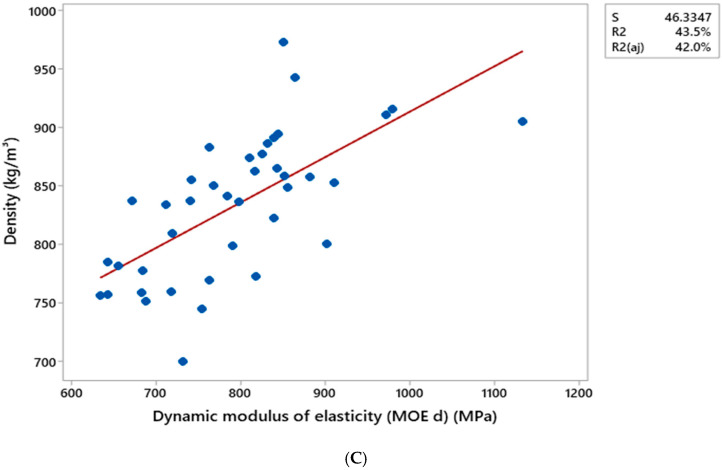
Dispersion matrix between the variables density, ultrasonic wave propagation velocity, and dynamic modulus of elasticity obtained from the ultrasonic wave propagation test using the pressing method, for the following combinations: (**A**) Ultrasonic Wave Velocity × Density, (**B**) Ultrasonic Wave Velocity × Modulus of Elasticity, and (**C**) Density × Modulus of Elasticity. The red line represents the straight line obtained from the linear regression of the data.

**Figure 8:**
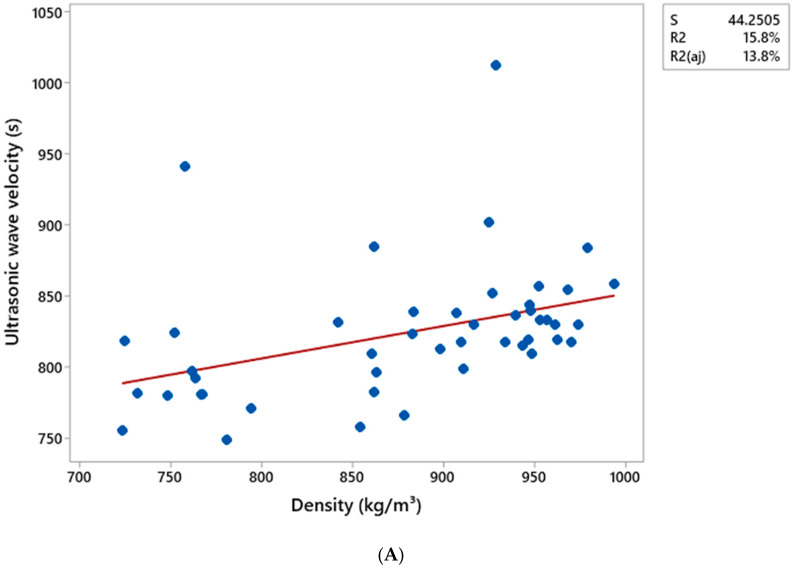

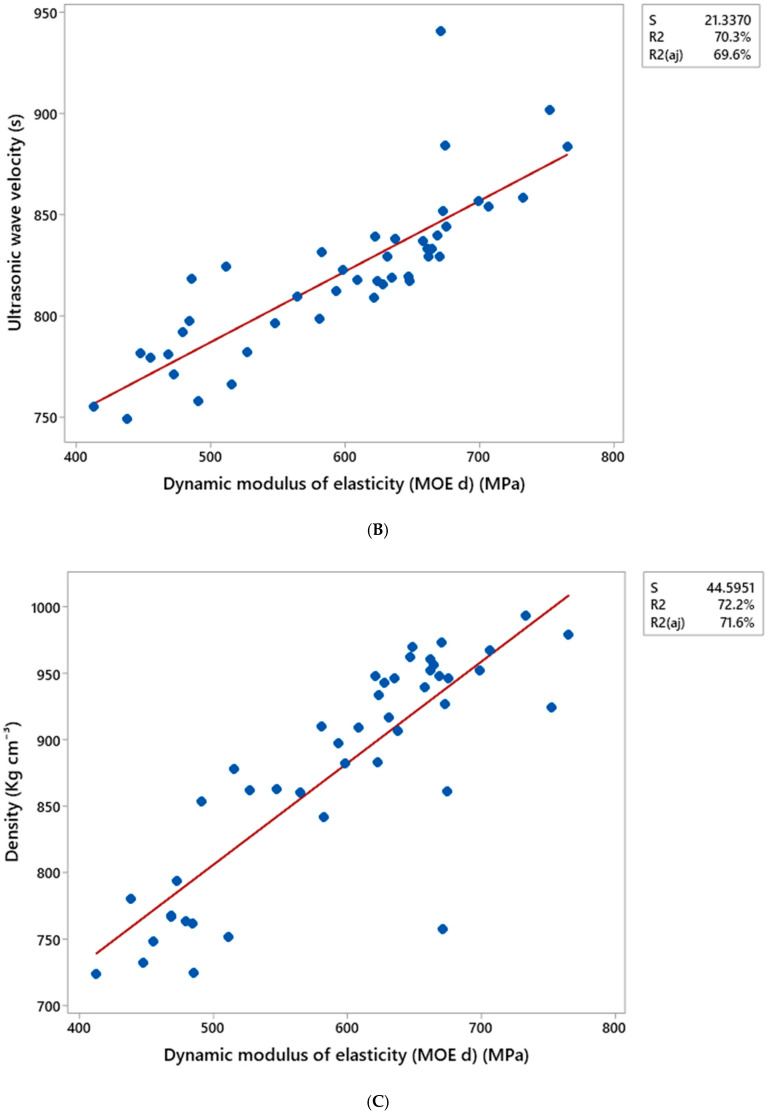
The dispersion matrix between the variables density, ultrasonic wave propagation velocity, and dynamic modulus of elasticity obtained through the ultrasonic wave propagation test using the extrusion method, for the following combinations: (**A**) Ultrasonic Wave Velocity × Density, (**B**) Ultrasonic Wave Velocity × Modulus of Elasticity, and (**C**) Density × Modulus of Elasticity. The red line represents the straight line obtained from the linear regression of the data.

Figure 7 and Figure 8 display the scatter plots for the combinations between the variable’s density (kg cm^−3^), ultrasonic wave speed (s), and elastic modulus (MPa), along with their respective coefficients of determination (R^2^). The letters A, B, and C represent the relationships between ultrasonic wave speed and density, ultrasonic wave speed and elastic modulus, and density and elastic modulus, respectively.

The correlations between ultrasonic wave speed and elastic modulus, as well as between density and elastic modulus, exhibited higher coefficients of determination (R^2^). For the composites manufactured by pressing, the R^2^ values were 68.2% and 43.5%, respectively, while for those produced by extrusion, the values were 70.3% and 72.2%.

These results are consistent with the Pearson correlation matrix, in which the relationship between ultrasonic wave velocity and density exhibited a lower correlation coefficient compared to the other relationships, also reflecting a lower R^2^ value.

## 4. Conclusions

1. The production process through pressing and extrusion demonstrated a significant difference in the static (MOE) and dynamic (MOE d) modulus of elasticity, as assessed by tensile and ultrasonic wave propagation tests, respectively. In contrast, the tests based on excitation wave propagation did not show significant differences in the obtained results.
2. The incorporation of coal particulate led to a reduction in the mechanical properties of the composites.
3. The reduction in mechanical properties does not rule out the use of these materials in components where the design can offset this limitation, by exploiting other characteristics such as lightness, thermal insulation, or chemical resistance. Furthermore, the use of this composite contributes to the valorization of waste and promotes a sustainable cycle, thereby reducing environmental impact.

## Figures and Tables

**Table 1:** Experimental design applied to the manufacturing of composites.

Production Process	Treatments	HDPE (%)	Particulate (%)
Pressing	T1	100	0
	T2	95	5
	T3	90	10
	T4	85	15
Extrusion	T1	100	0
	T2	95	5
	T3	90	10
	T4	85	15

**Table 2:** Comparative analysis of composite production methods (pressing and extrusion) based on maximum stress, using the Tukey test.

Production Process	Mean (Mpa) ^1^	Statistical Grouping
Pressing	15 ± 4.84	A
Extrusion	13.8 ± 2.94	A

^1^ Means followed by the same uppercase letter in the same row do not differ statistically from each other at the 5% probability level according to the Tukey test.

**Table 3:** Comparative analysis of the maximum tensile stress, as well as its respective deviations, determined by tensile testing and evaluated through the Tukey test.

Treatments ^2^	Pressing	Statisitcal Grouping ^1^	Extrusion	Statistical Grouping ^1^
T1	23.2 ± 4.48	A	18.8 ± 5.09	A
T2	13.2 ± 3.73	B	14.6 ± 2.66	AB
T3	12 ± 2.73	B	11 ± 2.06	B
T4	11.8 ± 1.78	B	10.6 ± 2.40	B

^1^ Means followed by the same uppercase letter in the same row do not differ statistically from each other at the 5% probability level according to the Tukey test. ^2^ PEAD: Fine particles: T1-100:0; T2-95:5; T3-90:10; and T4-85:15.

**Table 4:** Comparative analysis of composite production methods (compression molding and extrusion) based on the static elastic modulus (MOE), using Tukey’s test.

Production Process	Mean (Mpa) ^1^	Statistical Grouping
Pressing	287.14 ± 31.94	A
Extrusion	254.61 ± 57.66	B

^1^ Means followed by the same uppercase letter in the same row do not differ statistically from each other at the 5% probability level according to the Tukey test.

**Table 5:** Comparative analysis of the static modulus of elasticity (MOE), as well as their respective deviations, determined through tensile testing and assessed using the Tukey test.

Modulus of Elasticity (Mpa)				
Treatments ^2^	Pressing	Statisitcal Grouping ^1^	Extrusion	Statistical Grouping ^1^
T1	315.3 ± 27.7	A	306.6 ± 52.9	A
T2	278.0 ± 34.0	B	265.6 ± 60.6	AB
T3	276.7 ± 33.0	B	210.2 ± 10.0	B
T4	276.6 ± 15.1	B	209.2 ± 18.4	B

^1^ Means followed by the same uppercase letter in the same row do not differ statistically from each other at the 5% probability level according to the Tukey test. ^2^ PEAD: Fine particles: T1-100:0; T2-95:5; T3-90:10; and T4-85:15.

**Table 6:** Comparative analysis of composite production methods (pressing and extrusion) based on dynamic modulus of elasticity (MOE d), using the Tukey test.

Production Process	Mean (Mpa) ^1^	Statistical Grouping
Pressing	1904.7 ± 245.4	A
Extrusion	1873.5 ± 155.5	A

^1^ Means followed by the same uppercase letter in the same row do not differ statistically from each other at the 5% probability level according to the Tukey test.

**Table 7:** Comparative analysis of the dynamic modulus of elasticity (MOE d), as well as its respective deviations, determined through wave propagation testing and evaluated using the Tukey test.

Dynamic Modulus of Elasticity (Mpa)				
Treatments	Pressing	Statisitcal Grouping ^1^	Extrusion	Statistical Grouping ^1^
T1	2124.0 ± 153.1	A	2106.7 ± 75.7	A
T2	1997.3 ± 149.5	A	2028.2 ± 52.5	A
T3	1672.5 ± 149.7	B	1776.0 ± 52.7	B
T4	1634.0 ± 75.4	B	1731.0 ± 32.1	B

^1^ Means followed by the same uppercase letter in the same column do not differ statistically from each other at the 5% probability level according to the Tukey test.

**Table 8:** A comparative analysis of composite production methods (pressing and extrusion) based on density, ultrasonic wave velocity, and dynamic modulus of elasticity (MOE d) and their deviations, using a Tukey test.

Production Method	Density (kg/m^3^)	AE ^1^	Ultrasonic Wave Velocity (s) ^2^	AE ^1^	MOE (MPa) ^3^	AE ^1^
Pressing	838.1 ± 62	A	990 ± 69.8	A	791.2 ± 103	A
Extrusion	873.5 ± 75.4	A	827.5.0 ± 32.1	B	594.5 ± 81	B

^1^ AE: Statistical Clustering; means followed by the same uppercase letter in the same row do not differ statistically from each other at the 5% probability level according to the Tukey test. ^2^ Ultrasonic Waves (s) Vel.: velocity of ultrasonic waves. ^3^ MOE d (MPa): dynamic modulus of elasticity.

**Table 9:** Comparative analysis of the mean density, ultrasonic wave propagation velocity, and dynamic modulus of elasticity (MOE), as well as their respective deviations, determined by ultrasound testing and evaluated using the Tukey test.

Production ^4^ Method	Treatments	Density (kg/m^3^)	AE ^1^	Ultrasonic Wave Velocity (s) ^2^	AE ^1^	MOE (MPa) ^3^	AE ^1^
Pressing	T1	875.5 ± 71.8	A	1020.1 ± 108.8	A	819.3 ± 166	A
	T2	851.7 ± 45.9	A	979.4 ± 43.3	A	800.6 ± 92.9	A
	T3	850.8 ± 10.8	A	975.1 ± 33.4	A	796.3 ± 53.2	A
	T4	774.4 ± 42.6	B	974.1 ± 39.2	A	752.2 ± 68.5	A
Extrusion	T1	929.3 ± 35.4	A	845.9 ± 59.5	A	661.7 ± 41.8	A
	T2	927.7 ± 45.4	A	844.3 ± 22.69	A	643.9 ± 31.3	A
	T3	756.0 ± 21.5	B	806.9 ± 50.2	A	505.0 ± 68.5	B
	T4	881.2± 19.2	C	803.13 ± 28.7	A	561.2 ± 49.5	B

^1^ AE: Statistical Clustering; means followed by the same uppercase letter in the same row do not differ statistically from each other at the 5% probability level according to the Tukey test. ^2^ Ultrasonic Waves (s) Vel.: velocity of ultrasonic waves. ^3^ MOE (MPa): dynamic modulus of elasticity. ^4^ PEAD: Partículas carvão: T1-100:0; T2-95:5; T3-90:10; and T4-85:15.

**Table 10:** Pearson correlations between the variable’s density, ultrasonic wave propagation velocity, and dynamic MOE obtained through the ultrasonic wave propagation test using the pressing method.

Parameters	DE ^1^	VOU ^2^	MOE ^3^
DE	1	0.309	0.659
VOU	0.310	1	0.825
MOE	0.660	0.826	1

^1^ ρ = density (kg cm^−3^); ^2^ VOU = ultrasonic wave velocity (s); ^3^ MOE = modulus of elasticity (MPa).

**Table 11:** Pearson correlations between the variable’s density, ultrasonic wave propagation speed and dynamic MOE obtained by the ultrasonic wave propagation test using the extrusion method.

Parameters	DE ^1^	VOU ^2^	MOE ^3^
DE	1	0.121	0.741
VOU	0.397	1	0.723
MOE	0.850	0.839	1

^1^ ρ = density (kg cm^−3^); ^2^ VOU = ultrasonic wave velocity (s); ^3^ MOE = modulus of elasticity (MPa).

## Data Availability

The original contributions presented in this study are included in the article. Further inquiries can be directed to the corresponding author(s).
